# Lysosomal Rerouting of Hsp70 Trafficking as a Potential Immune Activating Tool for Targeting Melanoma

**DOI:** 10.2174/138161213804143644

**Published:** 2013-01

**Authors:** Kata Juhász, Roland Thuenauer, Andrea Spachinger, Ernő Duda, Ibolya Horváth, László Vígh, Alois Sonnleitner, Zsolt Balogi

**Affiliations:** 1 Center for Advanced Bioanalysis GmbH, Gruberstr. 40-42, A-4020 Linz, Austria; 2 Eukaryotic Molecular Biology Unit,; 3 Molecular Stress Biology Group, Institute of Biochemistry, Biological Research Center, Temesvari krt. 62, H-6701, Szeged, Hungary; 4 Institute of Medical Biology, University of Szeged, Somogyi B. u. 4., Szeged, Hungary

**Keywords:** Hsp70, transport to surface, release, lysosome, melanoma, immunotherapy.

## Abstract

Tumor specific cell surface localization and release of the stress inducible heat shock protein 70 (Hsp70) stimulate the immune system against cancer cells. A key immune stimulatory function of tumor-derived Hsp70 has been exemplified with the murine melanoma cell model, B16 overexpressing exogenous Hsp70. Despite the therapeutic potential mechanism of Hsp70 transport to the surface and release remained poorly understood. We investigated principles of Hsp70 trafficking in B16 melanoma cells with low and high level of Hsp70. In cells with low level of Hsp70 apparent trafficking of Hsp70 was mediated by endosomes. Excess Hsp70 triggered a series of changes such as a switch of Hsp70 trafficking from endosomes to lysosomes and a concomitant accumulation of Hsp70 in lysosomes. Moreover, lysosomal rerouting resulted in an elevated concentration of surface Hsp70 and enabled active release of Hsp70. In fact, hyperthermia, a clinically applicable approach triggered immediate active lysosomal release of soluble Hsp70 from cells with excess Hsp70. Furthermore, excess Hsp70 enabled targeting of internalized surface Hsp70 to lysosomes, allowing in turn heat-induced secretion of surface Hsp70. Altogether, we show that excess Hsp70 expressed in B16 melanoma cells diverts Hsp70 trafficking from endosomes to lysosomes, thereby supporting its surface localization and lysosomal release. Controlled excess-induced lysosomal rerouting and secretion of Hsp70 is proposed as a promising tool to stimulate anti-tumor immunity targeting melanoma.

## INTRODUCTION

As tumors are often resistant to conventional therapies, development of alternative approaches is needed. An emerging and promising clinical approach is immunotherapy, where the patient’s immune system is primed to fight cancer. The stress inducible heat shock protein 70 (Hsp70) has been evaluated as a special tool for immunotherapy as it was shown to play a role in anti-tumor immunity mediated by both innate and adaptive immune system [1-4]. Stress inducible Hsp70 is normally confined to the cytoplasm and exerts cytoprotective, anti-apoptotic functions [5, 6]. Surprisingly, Hsp70 has been found on various tumor cell surfaces, while the corresponding normal cells were surface negative [7-9]. Membrane-bound Hsp70 on the tumor cell surface has been shown to trigger cytolytic attack by natural killer cells [10]. Moreover, intracellular Hsp70 was reported to be released from cancer cells [11-17]. Once released, extracellular Hsp70 exerts a dual, “chaperokine” function acting as a chaperone and a cytokine. Released Hsp70, due to its chaperone function, has the capacity to present tumor antigens for the adaptive immune system. At the same time, extracellular Hsp70, even in the absence of immunogenic peptides, has been considered as a potent danger signal for the immune system [3, 18].

Low immunogenic melanoma is one of the most malignant tumors, which is prone to metastatize aggressively. It is often associated with poor therapeutic outcome, therefore causing a high mortality rate. Intriguingly, *in situ* killing of B16 melanoma using suicide gene transfer was associated with Hsp70 induction and triggered high immunogenicity [19]. Moreover, when tumor cells were transfected to overexpress Hsp70, they were equally as immunogenic as during necrotic cell death. Thus, Hsp70 expression could replace the necrotic mechanism in generating anti-tumor immunity [20]. Therefore we hypothesized that in addition to release of Hsp70 from dying cells [19] there may operate active transport mechanisms in B16 cells, which bring more Hsp70 to the surface or to the extracellular space. Although no experimental data are available about the transport mechanism of Hsp70 to the plasma membrane, it has been shown that Hsp70 can be released from live cells. Active mechanisms have been proposed to release Hsp70 in a soluble form via lysosomal endosomes or in a membrane-bound form via exosomes, largely depending on the cell type tested [12-14, 16, 21]. However, regulation of intracellular Hsp70 trafficking through specific compartments of the endolysosomal system remains to be explored. Here we investigated if excess Hsp70 in B16 cells could influence its surface localization and release, with an emphasis on understanding the trafficking mechanisms of Hsp70. We show endosomes as the major transport system bringing Hsp70 to the plasma membrane in B16 cells. Remarkably, excess Hsp70 was found to switch endosomal trafficking of Hsp70 to the lysosomes, bringing excess Hsp70 to the cell surface and to the extracellular space. 

## MATERIALS AND METHODS

### Cloning, transfection and cell culturing -

Mouse Hsp70 cDNA (Acc.No.: M35021) from pCR-Blunt/mHsp70 (a kind gift from K. Lisowska) was cloned into the tetracycline (TET) inducible mammalian expression vector pcDNA.4/TO (Invitrogen) at EcoRI and XbaI sites. To generate pcDNA.4/TO-mHsp70-mRFP1 the mouse Hsp70 sequence was amplified using the primers fwd GAGTCGA CGCCATGGCCAAGAACACG and rev GCCCGCGGTACCACCTCCTCGATGGTGGGTCCTGAG. The PCR product was cloned into the mRFP1 plasmid (kindly provided by H. Stockinger) at SalI and KpnI sites. The sequence of the fusion protein mHsp70-mRFP1 was subcloned into the vector pcDNA.4/TO at HindIII and NotI sites. All cloning (enzymes from Fermentas) was verified by sequencing. Inducible cell lines were generated by co-transfection of B16 (F10) cells with pcDNA.6/TR and either with empty pcDNA.4/TO or with pcDNA.4/TO-mHSP70 or pcDNA.4/TO-mHSP70-mRFP1 plasmids. Stable clones were selected by 60 µg/ml Zeocin (Invitrogen) and 8 µg/ml Blasticidin (InvivoGen). Cells were cultured in RPMI medium (Gibco) supplemented with 10 % FCS (Sigma), 2 mM L-glutamine (Gibco) and selection antibiotics. For experiments, cells transfected with empty or Hsp70 expressing vector were kept repressed (referred as “ctrl”) or induced by 2 µg/ml doxycycline (referred as “+Hsp70”), respectively. For “acute” or “chronic” Hsp70 production cells were induced for 16 h or 4 days, respectively. 

### Cellular fractionation -

Cellular fractions enriched in endosomes/ lysosomes were isolated essentially according to [22]. Briefly, 10^8^ cells were washed in PBS, scraped in homogenizing buffer (10 mM Tris, 250 mM sucrose (pH 7.0)) at 4 °C and disrupted with a Potter-Elvehjem homogenizer. The homogenate was pelleted at 1000x, 2000x and 4000x g. The obtained supernatant was treated with 0.1 mg/ml trypsin for 10 min at 37 °C followed by the addition of 0.4 mg/ml soybean trypsin inhibitor, 4 µg/ml aprotinin and leupeptin (Sigma) [23]. The diluted suspension was centrifuged for 2 min at 100000x g, at 4 °C (Sorvall, MX150), resulting in a pellet enriched in endosomes and lysosomes. Lysosomal fraction was isolated from the endolysosomal pellet by a 10 min hypotonic lysis using four times the pellet volume of distilled water. After centrifugation for 2 min at 100000x g, at 4 °C the lysosomal material remained in the supernatant that was concentrated for immunoblotting (Millipore). For isolating high purity soluble (cytosol) fractions cells were gently disrupted in the presence of protease inhibitors. 100000x g supernatants were centrifuged for 30 min at 400000x g, at 4 °C and supernatants were precipitated for immunoblotting [22]. 

### Intracellular and surface labeling -

Cells were detached with PBS (Gibco) containing 1 mM EDTA followed by 5 min incubation at 37 °C. Cells were then suspended in complete medium and pelleted. Samples for intracellular labeling were fixed in 1 % formaldehyde, permeabilized with 0.1 % Triton X-100 followed by washing with PBS (1 % FCS). 2x10^5^ cells were labeled with either 1.0 µg αHsp70-FITC (Stressgen) or isotype control (Sigma) for 1 h at room temperature. After washing samples were analyzed by flow cytometry with an excitation at 488 nm (BD FACSAria^TM^) gating for cell debris by FSC vs. SSC and detecting FITC signal using a bandpass filter of 530/30 nm. Samples for surface Hsp70 labeling were suspended in OPTI-MEM (Gibco) containing 1 % FCS. 1x10^5^ cells were labeled with either 1.0 µg a;-FITC, recognizing surface Hsp70 (Multimmune GmbH) [24] or with isotype control (Sigma) for 30 min at the indicated temperature and analyzed by flow cytometry. Debris and cells with damaged membranes were gated by FSC vs. SSC plotting and propidium-iodide (5 µg/ml, Sigma) exclusion (bandpass filter of 695/40 nm), respectively. Fluorescence values were corrected with the isotype controls, as appropriate. 

### Fluorescence quenching and transport to surface measurement -

Signal retained on the cellular surface of labeled samples was determined by fluorescence quenching [25, 26]. Cells were stained with αcmHsp70.1-FITC as described above, then FITC and propidium-iodide signals were detected in the channels of 530/30 nm and 576/26 nm with appropriate compensation, respectively. The same samples, which 0.1 % trypan blue (Sigma) was added to, were remeasured. The surface signal is reflected in the extent of reduction in the fluorescent values upon quenching. For measuring the rate of Hsp70 transport to the cellular surface the already membrane-localized Hsp70 molecules were neutralized by incubation with non-fluorescent αcmHsp70.1 at 37 °C for 10 min. Following removal of unbound αcmHsp70.1 Hsp70 transported freshly to the cellular surface was captured by incubation with αcmHsp70.1-FITC at 37 °C for 20 min then quantified by flow cytometry. Inhibitors were purchased from Biotrend (AACOCF_3_, 40 µM; bafilomycin A1, 200 nM; HELSS, 50 µM; ONO-RS-082, 10 µM) or from Sigma (brefeldin A, 6 µg/ml). Solvent controls were used as appropriate. Hyclone super low IgG supernatant (Thermo) containing 50 µg/ml anti-bis(monoacylglycero)phosphate (BMP) antibody (6C4) was kindly provided by J. Gruenberg [27]. Cells prior to experiment were incubated with either hyclone control or anti-BMP containing supernatant at 37 °C for 12 h. All data were corrected with the isotype controls, as appropriate. 

### Immunofluorescence microscopy -

Cells analyzed by microscopy were grown on glass bottom dishes (35 mm in diameter, Willco Wells BV). Cells were visualized by a custom designed instrument for large area fluorescence imaging (CytoScout^®^) based on an Axiovert 200 microscope (Zeiss) equipped with a 100x objective (alpha plan, N.A. 1.45) [28]. At an excitation wavelength of 488 nm FITC and propidium-iodide signals were monitored with emission filters 535/35 nm and 700/70 nm, respectively (AHF GmbH). Images were recorded with Coolsnap HQ cameras (Photometrics). Intensity scaling of representative images was adjusted with V++. For intracellular staining cells were fixed, permeabilized and labeled with either 1.0 µg αHsp70-FITC (Stressgen) or isotype control (Sigma) for 1 h at room temperature. After washing steps samples were scanned (1760x1760 µm^2^) for FITC signal by using the CytoScout^®^. Inhomogenous illumination was corrected based on background intensity profile (Matlab). Selected regions of images are shown with identical intensity scaling for image series of the same cell type. Color intensity scaling (Matlab, 0 - 1500 counts) is shown in each image to allow assessment for absolute intensities. 

### Dynamic co-localization experiment -

Adherent cells (2x10^5^) were labeled with 4 µg αcmHsp70.1-AlexaFluor647 (labeling kit from Invitrogen) at 37 °C for 30 min, followed by incubating cells with 25 nM LysoTracker Green (Invitrogen) for 5 min. At dual excitation (488 and 647 nm) the fluorophores were imaged with a 100x objective (apochromat, N.A. 1.40, Zeiss) using emission filters 535/50 nm and 700/75 nm. Dynamic localization of the fluorophores was followed by dual color real time fluorescence microscopy. Movies were inspected with ImageJ and overlay images of individual frames were created by Matlab subtracting background fluorescence. 

### Lysosensor imaging experiment -

Adherent cells (2x10^5^) were pulse-labeled with 4 µg αcmHsp70.1-AlexaFluor647 (labeling kit from Invitrogen) at 37 °C for 30 min. Cells washed were incubated at 37 ºC or at 43 ºC for 30 min prior to staining with 5 nM LysoSensor Green (Invitrogen) for 5 min. At dual excitation (488 and 647 nm) the fluorophores were imaged with a 100x objective (apochromat, N.A. 1.46, Zeiss) using the emission filters of 535/50 nm and 700/75 nm. Images were inspected with ImageJ and overlay images were created by Matlab subtracting background fluorescence. Cell boundaries were defined based on lysosensor fluorescence. Distribution of lysosensor intensity in all and Hsp70 labeled pixels was analyzed with Matlab applying manual thresholds for both lysosensor and AlexaFluor647 signals. An average distribution of normalized intensity histograms of single cells was calculated for each cell type and condition. For calculating the Manders’ colocalization coefficients for surface labeled Hsp70, a manual threshold defining lysosomes was set to normalized lysosensor intensities of 0.6. Average surface labeled Hsp70 signal/ pixel was assessed for low (0.0-0.6) and high (0.6-1.0) lysosensor intensity regions with Image J. No intensity difference between the non-lysosomal and lysosomal localized Hsp70 signals was observed. Therefore the number of Hsp70 positive pixels co-localizing with the high lysosensor intensity regions compared to the number of all Hsp70 pixels was used for quantification. 

### Hsp70mRFP imaging and low temperature blocking -

Localization of shortly (3 h) induced Hsp70-mRFP after a low temperature block applied (16 ºC, 3 h) and released (37 ºC, 30 min) was visualized with fluorescence microscopy. For simultaneous visualization of the endosomal system, cells were incubated with 0.1 mg/ml mouse transferrin-PacificBlue (Rockland, Invitrogen) while releasing the low temperature block. For simultaneous visualization of the lysosomal system, cells were loaded with 0.75 mg/ml dextran-CascadeBlue (Invitrogen) for 2 h and incubated for 16 h at 37 ºC prior to inducing Hsp70mRFP and subsequent temperature block. At dual excitation (405 and 514 nm) localization of transferrin-PacificBlue / dextran-CascadeBlue and Hsp70-mRFP was followed by using emission filters 464/25 nm and 625/25 nm, respectively. Images were recorded with a 100x objective (apochromat, N.A. 1.40, Zeiss). Overlay images of individual frames adjusted with appropriate threshold values were created with Matlab.

### Other analytical techniques -

Fixed and permeabilized cells, heat-treated samples and cellular fractions were tested by immunoblotting using anti-Hsp70 (Stressgen) [29], anti-transferrin (Rockland), anti-gp100 (Santa Cruz) and appropriate secondary antibodies (Sigma). b-N-acetylglucosaminidase assay was carried out according to the instructions (Sigma). Hsp70 ELISA (Stressgen) and lactate dehydrogenase (LDH) measurements (Promega) were carried out according to the protocols of manufacturers. 

### Statistical methods -

Experiments were carried out at least in triplicates (n ≥ 3) throughout all experimental data shown with mean ±SD. Other experiments (e.g. fractionation, immunoblotting or imaging) were done at least in duplicates. Data were analyzed by unpaired t-test. Multiple comparisons in datasets were probed with ANOVA. Significance is shown by asterisks, where *, ** and *** correspond to p= 0.01 - 0.05, p= 0.001 - 0.01 and p< 0.001, respectively. 

## RESULTS

### Intracellular excess of Hsp70 alters its cell surface appearance -

**Fig. (1):**
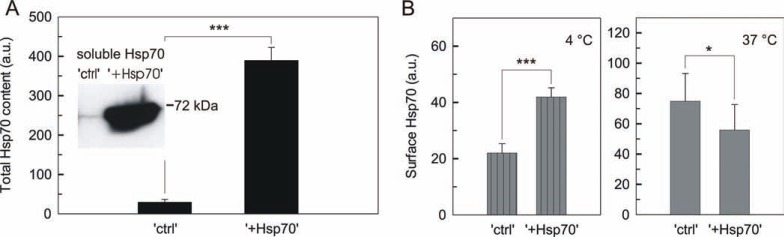
**Intracellular excess of Hsp70 alters its cell surface appearance** (**A**) Total Hsp70 content of B16 cells stably transfected with the TET on/off inducible system of either empty vector (ctrl) or Hsp70 expressing plasmid (+Hsp70). Hsp70 level of fixed and permeabilized cells was measured by flow cytometry (n ≥ 3; mean ±SD). (Insert) Representative Hsp70 immunoblotting for soluble fractions (cytosol) isolated from “ctrl” and “+Hsp70” cells. (**B**) Influence of intracellular expression on the cell surface appearance of Hsp70. Live cells were labeled at 4 °C or at 37 °C for 30 min and analyzed by flow cytometry (n ≥ 3 and n ≥ 7 for experiments at 4 °C and at 37 °C, respectively; mean ±SD). Note that intracellular and surface labeling were performed by using different antibodies.

Excess Hsp70 was achieved in B16 murine melanoma cells with a tetracycline-inducible system. Cells stably transfected with empty or Hsp70 expressing vector were kept repressed (referred as “ctrl”) or induced (referred as “+Hsp70”), respectively. Total Hsp70 content of “ctrl” and “+Hsp70” cells, quantified by flow cytometry after labeling permeabilized cells with a standard Hsp70 antibody (Fig. **1A**), reflected a high degree of exogenous protein production in “+Hsp70” cells. It is noteworthy that excess of Hsp70 also accumulated in the high purity soluble, cytosolic fraction of “+Hsp70” cells (Fig. **1A** insert). 

Surface Hsp70, which appeared on the cellular surface during time of labeling, was measured by flow cytometry. For this, a second type of antibody was used, which specifically recognizes also the membrane-localized Hsp70 [24]. In order to inhibit trafficking and measure the membrane concentration of Hsp70, cells were incubated with antibody at low temperature (4 °C). At these conditions “+Hsp70” cells showed a twofold higher membrane concentration compared to “ctrl” cells (Fig. **1B**). In order to preserve physiological-like trafficking, cells were incubated with antibody in a complete medium at 37 °C. Interestingly, at these conditions significantly less surface Hsp70 appeared on “+Hsp70” cells during time of labeling compared to “ctrl” cells. This reflected a lower turnover rate of surface Hsp70 in “+Hsp70” cells (Fig. **1B**).

### Transport of Hsp70 to the cell surface involves the endolysosomal system -

**Fig. (2):**
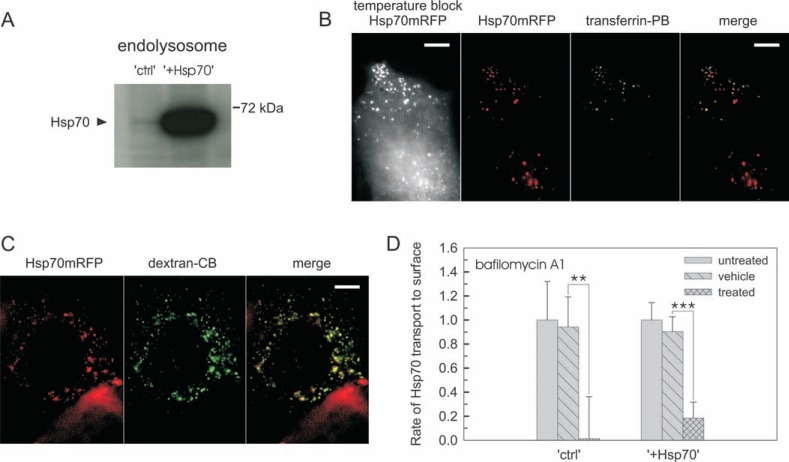
**Transport of Hsp70 to the cell surface involves the endolysosomal system** (**A**) Representative Hsp70 immunoblotting for endolysosomal fractions isolated from “ctrl” and “+Hsp70” cells. See also (Fig. 1S) for characterization of fractionation. (**B**) Visualization of endosomal localization of Hsp70-mRFP. While releasing a temperature block cells were labeled with transferrin-PacificBlue at 37 °C. Subcellular localization of Hsp70-mRFP is shown after releasing a temperature block (gray image, scale bar= 10 µm). Vesicular structures are displayed in red for Hsp70-mRFP and in green for transferrin-PacificBlue, respectively. A representative overlay image (merge) is shown with a scale bar of 10 µm. (**C**) Visualization of lysosomal localization of Hsp70-mRFP. Cells were loaded with dextran-CascadeBlue for 2 h and incubated for 16 h at 37 °C. Following a temperature block vesicular structures are displayed in red for Hsp70-mRFP and in green for dextran-CB, respectively. A representative overlay image (merge) is shown with a scale bar of 10 µm. (**D**) Transport of Hsp70 to the surface in “ctrl” and “+Hsp70” cells in the presence of the specific vacuolar ATPase (endolysosomal system) inhibitor. Cells were incubated with bafilomycin A1 at 37 °C for 30 min prior to measurements. Data are expressed in relation to the values of untreated cells (n ≥ 3; mean ±SD). For absolute values refer to (Fig. **4A**). (The color version of the figure is available in the electronic copy of the article).

The endolysosomal system has been implicated in Hsp70 trafficking [12-14, 16, 21]. To test if Hsp70 is transported to the surface through the endolysosomal system in B16 cells, cellular fractions enriched in endosomes and lysosomes were isolated and probed by immunoblotting. Hsp70 was detected in these cellular fractions of both “ctrl” and “+Hsp70” cells (Fig. **2A** and Supplementary Fig. **1**). However, a large excess of Hsp70 was observed in the endolysosomal system of “+Hsp70” cells as compared to “ctrl” counterparts. It is noteworthy that the extent of excess Hsp70 in “+Hsp70” cells was similar in the endolysosomal and cytosolic fractions (Fig. **2A** and Fig. **1A** insert). Endolysosomal localization of Hsp70 was further assessed by applying a temperature block [30, 31] in cells transfected with a fluorescently tagged Hsp70 (Hsp70mRFP). The temperature block caused Hsp70mRFP to accumulate in vesicular structures with partial co-localization between Hsp70mRFP and transferrin-labeled endosomes or dextran-labeled lysosomes (Fig. **2B** and **2C**, respectively). Next, the contribution of the endolysosomal system to Hsp70 transport to the surface was tested. In order for measuring the rate of this transport, Hsp70 already present in the membrane was neutralized with a non-fluorescent antibody, followed by labeling newly appearing Hsp70 with a fluorescent antibody (Fig. **2D**). When interfering with the function of the endolysosomal system by using a specific inhibitor of the vacuolar ATPases, bafilomycin A1 [31, 32], a detectable level of Hsp70 transport to the surface was nearly completely abolished in both “ctrl” and “+Hsp70” cells (Fig. **2D**). Altogether, these findings demonstrated that Hsp70 is transported to the cell surface via the endolysosomal system.

### Intracellular excess of Hsp70 switches its trafficking from the recycling endosomal to the lysosomal route -

**Fig. (3):**
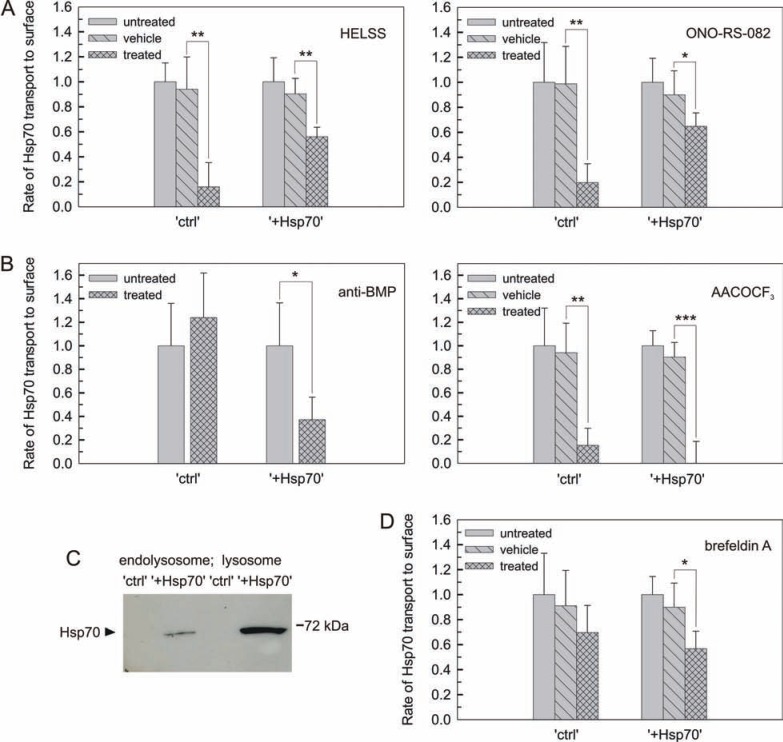
**Intracellular excess of Hsp70 switches its trafficking from the recycling endosomal to the lysosomal route** (**A**) Hsp70 transport to the surface in “ctrl” and “+Hsp70” cells in the presence of inhibitors of endosomal recycling. Cells were incubated either with HELSS or ONO-RS-082 at 37 °C for 30 min followed by the measurement. Data are expressed in relation to the values of untreated cells (n ≥ 3; mean ±SD). For absolute values refer to (Fig. 4A). (B) Hsp70 transport to the surface in “ctrl” and “+Hsp70” cells in the presence of lysosomal inhibitors. Cells were incubated with anti-BMP (blocking the lysosomal lipid, BMP) at 37 °C for 12 h or with AACOCF_3_ (inhibitor of endolysosomal trafficking) at 37 °C for 30 min prior to the measurement. Data are expressed in relation to the values of untreated cells (n ≥ 3; mean ±SD). For absolute values refer to (Fig. **4A**). (**C**) Representative Hsp70 immunoblotting for endolysosomal and lysosomal fractions isolated from “ctrl” and “+Hsp70” cells. See also (Fig. **1S**) for characterization of fractionation. (**D**) Hsp70 transport to the surface was measured in “ctrl” and “+Hsp70” cells incubated with brefeldin A (main inhibitory effect on the ER to Golgi trafficking) at 37 °C for 30 min. Data are expressed in relation to the values of untreated cells (n ≥ 3; mean ±SD). For absolute values refer to (Fig. **4A**).

To further dissect the involvement of the endolysosomal system in the transport of Hsp70 to the surface, the relative contribution of recycling endosomes and lysosomes was tested. First, cells were treated with specific inhibitors of Ca^2+^-independent phospholipase A_2_, which have been shown to block trafficking from the recycling endosomes to the plasma membrane without disturbing lysosomal trafficking [33, 34]. Transport of Hsp70 to the surface was nearly completely abolished when structurally unrelated inhibitors (HELSS and ONO-RS-082) were applied to “ctrl” cells. Surprisingly, the same inhibitor treatments resulted in a relatively small reduction in Hsp70 transport to the surface in “+Hsp70” cells (Fig. **3A**). These data indicated that Hsp70 transport to the surface occurs essentially via endosomes in “ctrl” cells, but other mechanisms play more fundamental roles in “+Hsp70” cells. 

Next, the role of lysosomal trafficking to Hsp70 transport to the surface was assessed with an antibody against the lipid bis(monoacylglycero)phosphate (BMP). This antibody abolishes Hsp70 interaction with the lysosomal membrane, hence interferes with lysosomal targeting of Hsp70 [27, 35]. Preincubation with this antibody was ineffective for “ctrl” cells. Unlike endosomal inhibitors, treatment with BMP antibody resulted in a sizable decrease in Hsp70 transport to the surface in “+Hsp70” cells (Fig. **3A** and **3B**). Alternatively, cells were challenged with AACOCF_3_, an inhibitor of both Ca^2+^-independent and Ca^2+^-dependent phospholipase A_2_. This compound interferes with both endosomal and lysosomal fusion events at the plasma membrane and can be used for blocking lysosomal secretion [33, 34]. Remarkably, when cells were incubated with AACOCF_3_, Hsp70 transport to the surface was not further inhibited in “ctrl” cells as compared to the effect of endosomal inhibitors, but was completely abrogated in “+Hsp70” cells (Fig. **3A** and **3B**). These data showed that the major pathway of Hsp70 transport to the surface was shifted towards the lysosomal system in “+Hsp70” cells. This was further supported by the fact that a substantial enrichment of Hsp70 in the lysosomal vs. endolysosomal fraction was detected in “+Hsp70” cells (Fig. **3C** and Supplementary Fig. **1**). 

Hsp70 is believed to be released to the extracellular space through a non-classical vesicular pathway [12-14, 16, 21]. To test whether Hsp70 transport to the surface bypasses the classical route, the transport was measured in the presence of brefeldin A. Brefeldin A inhibits protein trafficking at the endoplasmic reticulum-Golgi juncture and the trans-Golgi network. In addition, it has been reported to interfere with lysosomal trafficking leaving endosomal recycling unaffected [36]. This inhibitor had no significant effect on Hsp70 transport to the surface in “ctrl” cells, but some reduction was detected in “+Hsp70” cells (Fig. **3D**). This reduction could be due to the side-effect of brefeldin A on lysosomal trafficking of Hsp70 in “+Hsp70” cells (see Fig. **3B** and **3C**). Therefore these data suggested that Hsp70 is delivered via a non-conventional mechanism to the endolysosomal system *en route* to the plasma membrane. 

### Intracellular excess of Hsp70 supports its membrane trafficking upon heat stress -

**Fig. (4):**
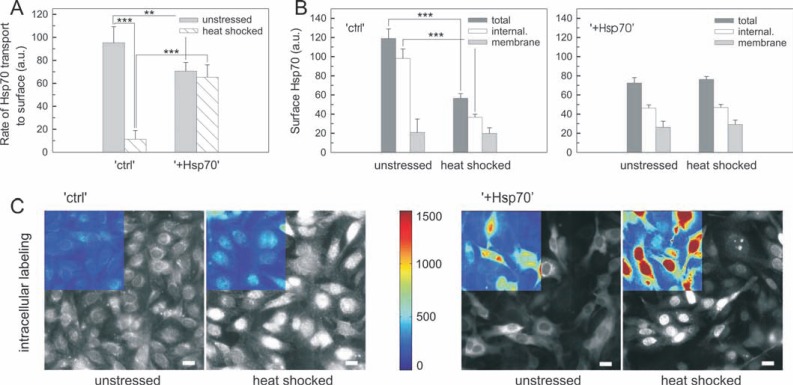
**Initial excess of intracellular Hsp70 supports its membrane trafficking upon heat stress** (**A**) Hsp70 transport to the cellular surface in unstressed or heat shocked cells. “ctrl” and “+Hsp70” cells were incubated either at 37 °C or at 43 °C for 30 min, then all samples were shifted to 37 °C for subsequent transport measurements (n = 4; mean ±SD). (**B**) Surface localized and internalized Hsp70 in unstressed or heat shocked cells. “ctrl” and “+Hsp70” cells were incubated either at 37 °C or at 43 °C for 30 min, then samples were shifted to 37 °C for subsequent labeling for 30 min. Surface and internalized signals of viable cells were measured by flow cytometry (n = 4; mean ±SD). Fluorescent signal retained in the cellular membrane was quenched by trypan blue. (**C**) Relocalization of Hsp70 in “ctrl” and “+Hsp70” cells upon heat stress. Adherent cells were kept at 37 °C as unstressed control or heat shocked at 43 °C for 30 min. Representative selection of large area fluorescence image of intracellular Hsp70 staining is shown (scale bar= 20 µm). Notice different intensity scaling for the gray image series of different cell types. Absolute intensities can be assessed in color images.

It has been shown that high temperature stress or heat analogue membrane fluidization could increase membrane concentration of Hsp70 [7, 11, 14, 17, 29, 37]. To reveal whether and how the trafficking of Hsp70 changes in B16 cells upon heat stress we focused on the initial stages of stress response. The rate of Hsp70 transport to the surface was found significantly lower in “+Hsp70” cells at unstressed conditions (Fig. **4A**). However, the transport rate was dramatically reduced in “ctrl” and remained unaffected in “+Hsp70” cells upon heat stress (Fig. **4A**). All surface Hsp70 present at the membrane during time of antibody labeling was measured as described before (see Fig. **1B**). In “ctrl” but not in “Hsp70” cells there was a notable reduction in the amount of surface Hsp70 appearing at the membrane upon heat stress (Fig. **4B**). We further examined the change in the fraction of labeled Hsp70 retained or recycled to the surface by assessing extracellular fluorescence quenching. Using the membrane impermeant dye trypan blue [25, 26], quenchable or unquenchable signals reflected membrane or internalized Hsp70, respectively. In “ctrl” cells the internalization of Hsp70 was greatly inhibited upon heat stress, meanwhile the membrane component remained unaltered as reflected by the quenchable signal (Fig. **4B**). In “+Hsp70” cells neither the internalized nor the membrane signals were affected by heat stress (Fig. **4B**). These data showed that intracellular excess of Hsp70 prevented heat-induced inhibition of Hsp70 transport to the cellular surface and internalization of surface Hsp70. 

When cells are challenged by heat stress, Hsp70 is required for supporting protein machineries in refolding and could also translocate into the nuclei of heat stressed cells [38, 39]. Since heat stress can cause a substantial depletion of the cytosolic Hsp70 pool, we tested the nuclear translocation of Hsp70 in “ctrl” and “+Hsp70” cells (Fig. **4C**). As shown, both endogenous and exogenous Hsp70 displayed a diffuse pattern of intracellular and perinuclear staining in unstressed cells. Heat stress was clearly associated with a massive translocation of Hsp70 from the cytosol to the nucleus. Nevertheless, in “+Hsp70” cells a considerable excess of Hsp70 was still present outside the nuclei (see (Fig. **4C**), color inserts at equal scaling for heat stressed “ctrl” vs. “+Hsp70” cells). Moreover, after initial stages of heat stress response, trafficking of surface Hsp70 appeared to recover in heat stressed “ctrl” cells (Supplementary Fig. **2A**). In fact, kinetics of the recovery was paralleled with the rate of *de novo* Hsp70 synthesis (Supplementary Fig. **2B**). Therefore the above data showed that trafficking of surface Hsp70 is controlled by the cytosolic pool of Hsp70. 

### Intracellular excess of Hsp70 drives surface Hsp70 towards lysosomes for heat-triggered secretion -

**Fig. (5):**
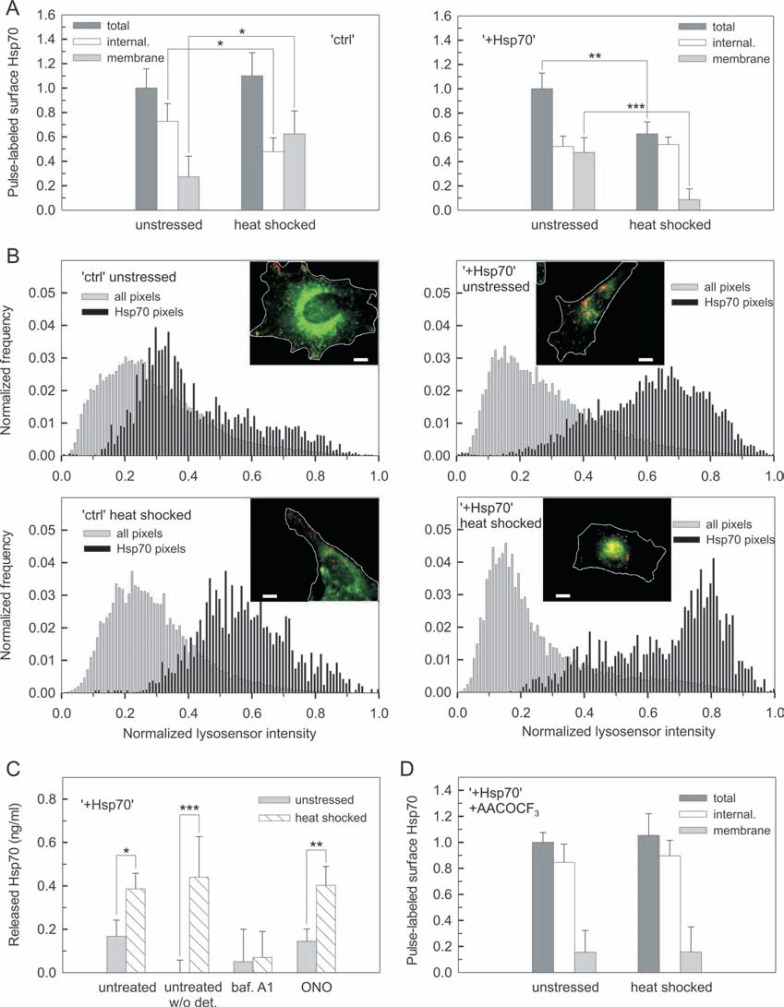
**Intracellular excess of Hsp70 drives surface Hsp70 towards lysosomes for heat-triggered secretion** (**A**) Pulse-chase experiment for tracking surface Hsp70 in “ctrl” and “+Hsp70” cells. Cells were pulse-labeled with antibody at 37 °C for 30 min, then incubated at 37 °C (unstressed) or at 43 °C (heat shocked) for 30 min prior to fluorescence quenching experiment (n ≥ 3; mean ±SD). (**B**) Lysosomal targeting of internalized surface Hsp70 in “ctrl” and “+Hsp70” cells. Adherent cells were pulse-labeled with antibody at 37 °C for 30 min and incubated at 37 °C or at 43 °C for 30 min prior to staining with lysosensor. (Inserts) Localization of labeled surface Hsp70 (red) and the lysosomal marker (green) is shown in representative overlay images. Cell boundaries were defined based on lysosensor fluorescence (scale bar= 10 µm). Average distribution of lysosensor intensity in all or Hsp70 positive pixels is shown in histogram display for each cell type and condition (n = 4-8). See also (Fig. **3S**) for further analysis shown. Manders’ coefficients for co-localization between surface labeled Hsp70 and lysosomes are 0.188 for “ctrl unstressed”, 0.558 for “+Hsp70 unstressed”, 0.405 for “ctrl heat shocked” and 0.657 for “+Hsp70 heat shocked” cells. (**C**) Release of Hsp70 from “+Hsp70” cells. Adherent cells were incubated in the absence or presence of inhibitors at 37 °C for 30 min, then either kept at 37 °C or exposed to 43 °C for 30 min. Cells were allowed to recover at 37 °C for 30 min before measuring Hsp70 concentration of the supernatants (n ≥ 3; mean ±SD). All samples were solubilized with detergent treatment before measurements unless stated otherwise. Notice that release was not detectable from “ctrl” cells. See also (Fig. **4S**) for LDH activities of supernatants. (**D**) Effect of inhibiting lysosomal secretion on the loss of pulse-labeled surface Hsp70 from heat shocked “+Hsp70” cells. Cells were pulse-labeled with antibody at 37 °C for 30 min and then incubated at 37 °C or at 43 °C for 30 min in the presence of AACOCF3. See also (Fig. **5S**) for experiment in the presence of ONO-RS-082. Total, internalized and membrane signals were assessed in fluorescence quenching experiments (n = 4; mean ±SD). (The color version of the figure is available in the electronic copy of the article).

Our results indicated that endosomes and lysosomes are compartments for Hsp70 transport to the surface. We speculated that upon internalization (see Fig. **4B**) the surface Hsp70 is targeted to the endolysosomal compartments, thereby establishing a trafficking circle. In order to investigate this hypothesis, surface Hsp70 was labeled with antibody and was allowed to get internalized while following its co-localization with the endolysosomal marker lysotracker. We observed a clear co-localization of the internalized protein with acidic lysotracker-positive compartments of punctuate pattern. Moreover, time resolved images revealed co-movement of endocytosed surface Hsp70 with endolysosomes (Supplementary movie **1**). Similar results were obtained when the Fab fragment of the antibody was used for labeling (data not shown). Next, internalization of pulse-labeled surface Hsp70 was quantified in unstressed and heat stressed cells by fluorescence quenching experiments. Note that membrane and internalized components can be assessed right after pulse labeling as unstressed values shown in (Fig. **4B**). In “ctrl” cells heat stress increased the quenchable membrane signal, which was paralleled with a decrease in the unquenchable internalized component (Fig. **5A**). These findings pointed to an increased recycling of internalized surface Hsp70 in heat stressed “ctrl” cells. In contrast, there was a considerable loss of the pulse-labeled surface Hsp70 in heat stressed “+Hsp70” cells. Apparently, loss of the pulse-labeled signal was associated with a nearly complete loss of the membrane component, while the internalized signal remained unaltered (Fig. **5A**). 

Cancer cells have been shown to take up recombinant Hsp70 targeted to the lysosomes, where Hsp70 is thought to play a functional role [5, 35]. Therefore, a pH sensitive marker of lysosomes (lysosensor) was used to further resolve localization and targeting of internalized surface Hsp70. Following pulse labeling of surface Hsp70 and lysosensor labeling, all pixels through the cell image were analyzed for lysosensor intensity (Fig. **5B**, gray histograms). Most of the pixels displayed rather low lysosensor values and only a small fraction corresponding to more acidic compartments showed high values. Distribution of lysosensor intensities of pixels positive for pulse-labeled surface Hsp70 was also analyzed (Fig. **5B**, black histograms). Comparison of the two histograms allowed estimation of the extent of lysosomal targeting of internalized surface Hsp70. In unstressed “ctrl” cells no preferential accumulation of labeled surface Hsp70 was observed in high lysosensor intensity regions. In contrast, a large fraction of labeled surface Hsp70 in unstressed “+Hsp70” cells appeared to localize to more acidic regions characterized with high lysosensor values. Labeled surface Hsp70 moved towards regions of higher lysosensor intensities in both cell types upon heat stress, but the majority of Hsp70 still remained in the regions of intermediate lysosensor intensities in “ctrl” cells (Fig. **5B** and Supplementary Fig. **3**). These measurements revealed a preferential lysosomal targeting of internalized surface Hsp70 in “+Hsp70” cells. 

The loss of pulse-labeled surface Hsp70 suggested that internalized surface Hsp70 may be released through the endolysosomal system. First, we explored this possibility by testing the release of Hsp70 from B16 cells. There was no detectable level of released Hsp70 measured at any condition for “ctrl” cells (data not shown). In contrast, a low level of Hsp70 was detectable in the supernatant of “+Hsp70” cells under unstressed conditions (Fig. **5C**). This may be attributed to a fraction of passively released Hsp70, given the presence of measurable lactate dehydrogenase (LDH) levels (Supplementary Fig. **4**) which is an indicator of passive release. Although detergent treatment did not make a significant difference ((Fig. **5C**) untreated unstressed vs. untreated unstressed w/o detergent samples), active secretion of Hsp70 via exosomes at basal conditions may also not be excluded [12, 16, 40]. Upon heat stress a 2.5-fold increase in released Hsp70 concentration was observed for “+Hsp70” cells (Fig. **5C**). Since no increase in the LDH content was measured in the same samples, the stress-induced release of Hsp70 could be attributed to active mechanisms (Supplementary Fig. **4**). To explore the mechanism of active release of Hsp70 further, we tested the involvement of different compartments. Bafilomycin A1, a specific inhibitor of endolysosomal targeting, efficiently blocked the stress-induced active release of Hsp70 from “+Hsp70” cells. In contrast, inhibiting the endosomal pathway with ONO-RS-082 did not affect release of Hsp70 from “+Hsp70” cells (Fig. **5C** and Supplementary Fig. **4**). Note that Hsp70 was found to be released in a soluble form upon heat stress as detergent solubilization of the sample did not make a difference (Fig. **5C**). 

Our data supported a strong correlation between lysosomal targeting of surface Hsp70, release of Hsp70 through the lysosomes and loss of surface Hsp70 upon heat stress. We further tested the fate of pulse-labeled surface Hsp70 in the presence of AACOCF_3._ Blocking active lysosomal secretion with this inhibitor completely prevented “+Hsp70” cells from loss of surface Hsp70 upon heat stress (Fig. **5D**). As a control we used ONO-RS-082, an inhibitor of the endosomal pathway, which did not block the loss of surface Hsp70 (Supplementary Fig. **5**). Therefore, part of the pulse-labeled surface Hsp70 was targeted to lysosomes and released from heat stressed “+Hsp70” cells.

### Stress-induced active release and loss of surface Hsp70 require acute excess of intracellular Hsp70 -

**Fig. (6):**
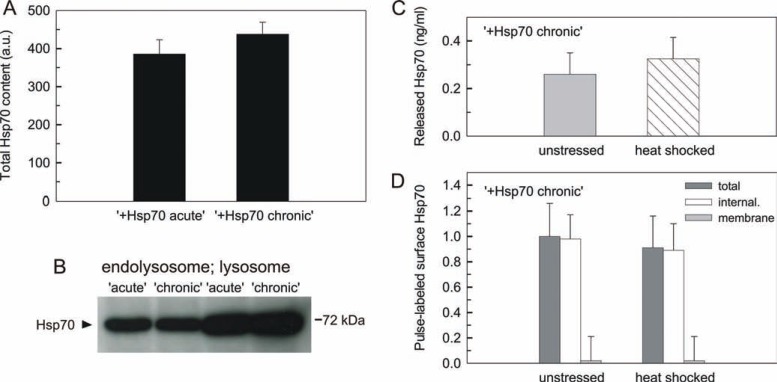
**Chronic excess Hsp70 does not support active release and loss of surface Hsp70 upon heat stress** (**A**) Total Hsp70 content of B16 cells expressing Hsp70 acutely or chronically. Cells stably transfected with the inducible Hsp70 expressing system (“+Hsp70”) were induced either for 16 h (“acute”) or for 4 days (“chronic”). Total Hsp70 level was measured by flow cytometry (n = 4; mean ±SD). (**B**) Representative Hsp70 immunoblotting for endolysosomal and lysosomal fractions isolated from “+Hsp70” cells with acute or chronic excess Hsp70. See also (Fig. **1S**) for characterization of fractionation. (**C**) Loss of stress-induced active release from chronically induced “+Hsp70” cells. Adherent cells were either kept at 37 °C or exposed to 43 °C for 30 min. Cells were allowed to recover at 37 °C for 30 min before Hsp70 concentration of solubilized supernatants was measured (n = 4; mean ±SD). Notice that there was no change in LDH activity upon heat shock. (**D**) Loss of releasing surface Hsp70 from chronically induced “+Hsp70” cells upon heat stress. Cells were pulselabeled at 37 °C for 30 min and then incubated at 37 °C or at 43 °C for 30 min prior to fluorescence quenching experiments (n ≥ 3; mean ±SD).

Transiently expressed Hsp70 analyzed in the above experiments and referred as “acute” has been reported to trigger a more efficient immune activation as compared to a constitutive protein production [41]. Surface and released Hsp70, both with immune stimulatory potential, were therefore also tested in “+Hsp70” cells producing exogenous protein for 4 days (referred as “chronic”). Total Hsp70 levels of “acute” and “chronic” cells showed no significant difference (Fig. **6A**). Accumulation of Hsp70 in the lysosomal fraction of “chronic” cells was also similar to that observed for the “acute” cells (Fig. **6B**). In addition, no difference was seen in surface Hsp70 measurements upon prolonged production of intracellular excess of Hsp70 (data not shown). However, neither active release nor loss of surface Hsp70 were detectable from heat-triggered “chronic” “+Hsp70” cells (Fig. **6C** and **6D**). Therefore, active release and stress-induced loss of surface Hsp70 appeared to require acute excess of intracellular Hsp70. 

## DISCUSSION

Hsp70-based cancer immunotherapy is an emerging and promising approach with documented preclinical progress in the treatment of aggressive tumors such as melanoma. Tumor specific approaches are based on the immune stimulatory function of unusual surface localized and extracellular Hsp70 [1-4, 42]. Despite the therapeutic potential of facilitating surface localization and release, trafficking mechanisms and routes of Hsp70 to the cell surface and to the extracellular space still remain poorly understood. To examine the principles of Hsp70 trafficking we have used an established melanoma model cell line, B16 [43]. Breaking immune tolerance *in vivo *through up-regulation of Hsp70 in the low immunogenic B16 cells [19, 44] made this cell model a valuable experimental tool to study mechanism of Hsp70 trafficking and potential impact of elevated Hsp70 levels on its localization hence on its immune activating properties. 

Our data strongly suggest a vesicular mechanism for transport of Hsp70 to the cell surface and to the extracellular space in B16 melanoma cells. The endolysosomal system has been implicated in Hsp70 trafficking in various cell types [5, 12-14, 16, 21]. In support of this, we found Hsp70 in the endosomal/lysosomal fraction of B16 cells. In agreement with former suggestions [16, 40] targeting Hsp70 to the vesicular system appeared to bypass the classical endoplasmic reticulum-Golgi secretory pathway in B16 cells. Furthermore, detailed analysis revealed that Hsp70 trafficking is essentially confined to the endosomal system and there is no detectable Hsp70 released from these cells. Introducing an excess of Hsp70, however, triggered a series of remarkable changes in Hsp70 trafficking and localization. First, a cytosolic excess was associated with rerouting of Hsp70 trafficking from an endosomal to a lysosomal pathway. Second, this was paralleled with accumulation of Hsp70 in the lysosomal system. Third, lysosomal rerouting reduced contribution of fast endosomal recycling, hence slowing down apparent turnover of surface Hsp70. In addition*,* excess Hsp70 in the lysosomal system supplied an elevated membrane concentration of Hsp70. Fourth, lysosomal excess allowed active release of soluble Hsp70 from B16 melanoma cells through these compartments. Lysosomal accumulation of Hsp70 may operate as part of a mechanism to remove non-needed excess protein via digestion or release [16, 17, 40, 45]. Nevertheless, because of an active role in the lysosomal system [5, 35, 46] Hsp70 is clearly distinguishable from other cargos destined for lysosomal digestion. In support of this, we showed that an acute excess of cytosolic Hsp70 gave rise to cell surface and extracellular localization of Hsp70 through lysosomal rerouting. 

Cancer cells are often challenged by the tumor microenvironment or therapeutic treatments. Various kinds of stress have been shown to influence surface localization and release of Hsp70 [7, 11, 14-17, 37, 24, 47]. Here we have examined effects of the therapeutically used hyperthermia [43] on Hsp70 trafficking. Upon heat stress there is an immediate need for Hsp70 for supporting cellular functions [39]. Strikingly, stress-induced temporary shortage in the “free” pool of Hsp70 [38] was accompanied with a severe depletion of trafficking of surface Hsp70 in B16 melanoma cells. In contrast, prior excess of Hsp70 protected trafficking of surface Hsp70 from heat-triggered depletion. Moreover, recovery of the original trafficking in “ctrl” cells correlated with *de novo* Hsp70 production. Therefore, dynamics of surface Hsp70 appeared to be tightly controlled by the available cytosolic Hsp70 pool. In heat stressed “ctrl” cells, lack of excess Hsp70 was counterbalanced by increased endosomal recycling of internalized surface Hsp70. In contrast, prior excess of Hsp70 facilitated removal of internalized surface Hsp70 via lysosomal secretion upon heat stress. This novel pathway resembles the previously proposed release mechanism of internalized surface Hsp70 via exosomes [13], possibly reflecting a melanoma specific fate through the lysosomes. Releasing surface Hsp70, because of a potential co-presentation of chaperoned surface proteins [9, 24], may represent a highly efficient mechanism to stimulate the immune system [3, 13]. 

Up-regulating Hsp70 has been shown to increase immunogenicity of B16 tumors and Ge human melanoma cells in animal and cell culture experiments, respectively [19, 41]. Moreover, facilitation of surface localization or release of Hsp70 has been shown to correlate with increased immunogenicity of several tumor cell types tested in mice or at *in vitro* conditions [19, 47-50] These observations together with our findings indicate that Hsp70 overexpression in B16 cells could generate an excellent immune activating milieu through an elevated level of surface and released Hsp70. Furthermore, we showed that B16 melanoma cells chronically expressing excess Hsp70 lost their ability to release Hsp70 through active transport mechanisms. In line with this, constitutive excess of Hsp70 expressed in melanomas has been found less immunogenic [19, 41, 44], a phenomenon that has been attributed to cellular acclimation to non-needed excess of Hsp70 [41, 51, 52]. Accordingly, acute excess-mediated active release mechanisms of Hsp70 appear to contribute to a more efficient immune stimulation against melanoma cells. 

Human melanomas with often up-regulated Hsp70 expression [53-55] may resemble B16 cells chronically expressing an excess of Hsp70 in terms of high cytosolic and lysosomal Hsp70 levels as well as a reduced ability to release Hsp70 (our unpublished data). In accordance, melanoma cells are usually low immunogenic irrespective of their basal level of Hsp70 [44, 56]. Increasing immunogenicity of melanoma cells, frequently resistant to conventional therapies [57] would be therefore a desirable strategy. This would, however, require assuring active transport mechanisms of Hsp70 in the survival population. An acute excess achieved by repeated Hsp70 gene transfer alone or in combination with hyperthermia proved to kill large established melanoma or to arrest tumor growth in mice, respectively [44, 58]. Alternatively, an acute excess of Hsp70 induced by different therapies of human melanomas [1, 43] may be utilized to stimulate the immune system against resistant tumor populations. In support of this idea, loco-regional hyperthermia as an adjuvant to radiotherapy was found effective in clinical trials for melanoma [59]. Finally, membrane perturbing drugs have been emerging as promising tools to stimulate Hsp expression and transport [29, 37, 60-62]. The Hsp co-inducer BGP-15 has been recently shown to inhibit the rapid HSF1 acetylation during the early phase of heat stress, thereby promoting a prolonged heat shock response. Meanwhile the same compound activated Rac1, a Rho family member of small GTPases in B16 melanoma cells [63]. Development of clinically effective approaches targeting Hsp70 trafficking requires further exploring of novel drug candidates and treatment strategies. 

## CONCLUSION

**Fig. (7):**
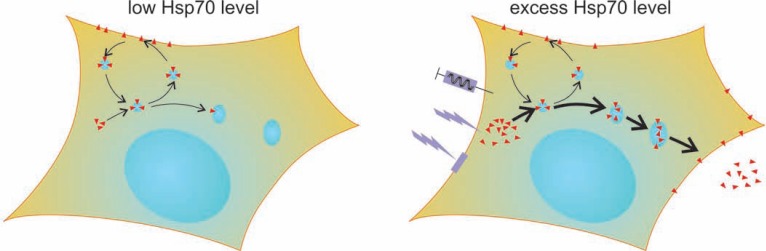
**Proposed schematic model for facilitating surface localization and release of the immune stimulatory Hsp70 in melanoma cells via excessinduced lysosomal rerouting and secretion** In low Hsp70 expressing cells trafficking of Hsp70 (red triangles) is mediated by endosomes. Up-regulating cytosolic Hsp70 level diverts Hsp70 trafficking from the endosomal to the lysosomal route and accumulates Hsp70 in the lysosomal system. Acute but not chronic excess of Hsp70 and triggering lysosomal secretion can give rise to surface localization and release of Hsp70. An acute excess of Hsp70 may be achieved repeatedly by gene transfer, stress or membrane perturbing drugs. Active lysosomal secretion of an acute excess of Hsp70 may be facilitated by stress or membrane perturbing drugs. Development of approaches that up-regulate the expression and target the secretory pathway of Hsp70, is of therapeutic potential to stimulate the immune system against melanoma.

Altogether, we demonstrate novel principles of a vesicular trafficking system bringing excess intracellular Hsp70 to facilitate its immune stimulatory role, and propose that controlled stimulation of this trafficking machinery may improve the immunotherapy of melanoma (Fig. **7**).
