# Supplementary material for: Lysosomal Rerouting of Hsp70 Trafficking as a Potential Immune Activating Tool for Targeting Melanoma
Source: Curr Pharm Des. 2013 Jan;19(3):430–40. doi: 10.2174/138161213804143644 (PMC3531874; doi:10.2174/138161213804143644)
Supplement: Supplementary file 1 [file CPD-19-430_SD1.zip › 2013_00000019_00000003_0009B/9-Supplimentary-MS3.pdf]

Supplementary Material

Lysosomal Rerouting of Hsp70 Trafficking as a Potential Immune Activating Tool for Targeting Melanoma

Kata Juhász, Roland Thuenauer, Andrea Spachinger, Ernő Duda, Ibolya Horváth, László Vígh, Alois Sonnleitner and Zsolt Balogi

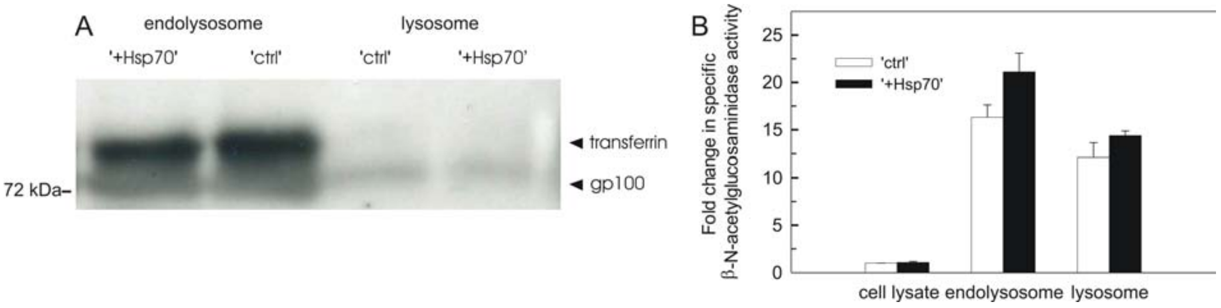

Fig. (1S). Characterization of cellular fractions For characterization, "ctrl" and "+Hsp70" cells were loaded with transferrin. Proteolytically digested fractions enriched in endolysosomes or lysosomes were isolated. (A) Endosomal and melanosomal contamination in the lysosomal fractions were assessed by immunoblotting for transferrin or gp100, respectively. (B) Hydrolase activities, characteristic for melanosomes and lysosomes [64] were assayed for endolysosomal and lysosomal fractions. Notice a 12-20-fold enrichment in specific activities compared to the total cell lysates.

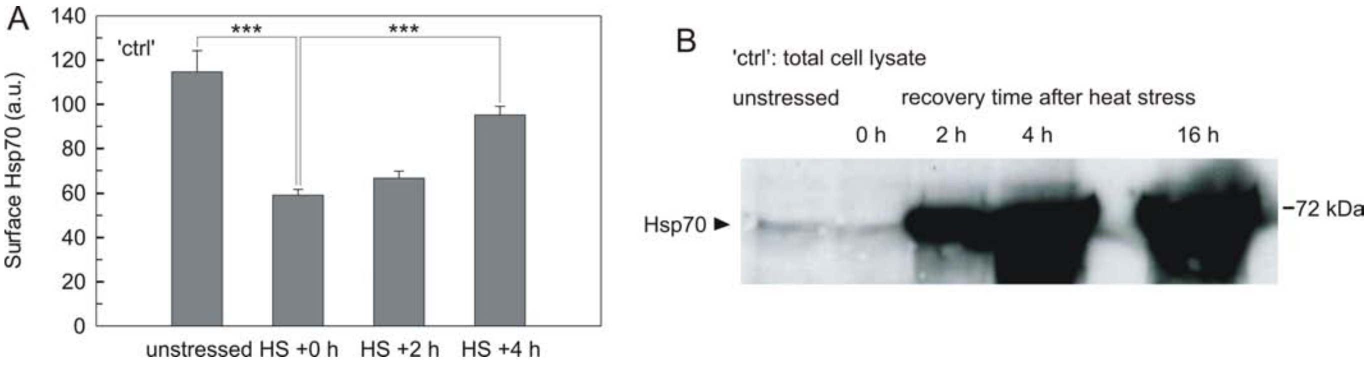

Fig. (2S). Recovery of surface Hsp70 appearance in heat shocked "ctrl" cells (A) Surface and (B) total level of Hsp70 in "ctrl" cells after heat stress (HS). Adherent cells were either kept at 37 ºC or incubated at 43 ºC for 30 min and allowed to recover at 37 ºC for 0, 2 or 4 h. All samples were tested for surface Hsp70 by flow cytometry and probed by immunoblotting for total Hsp70 content.

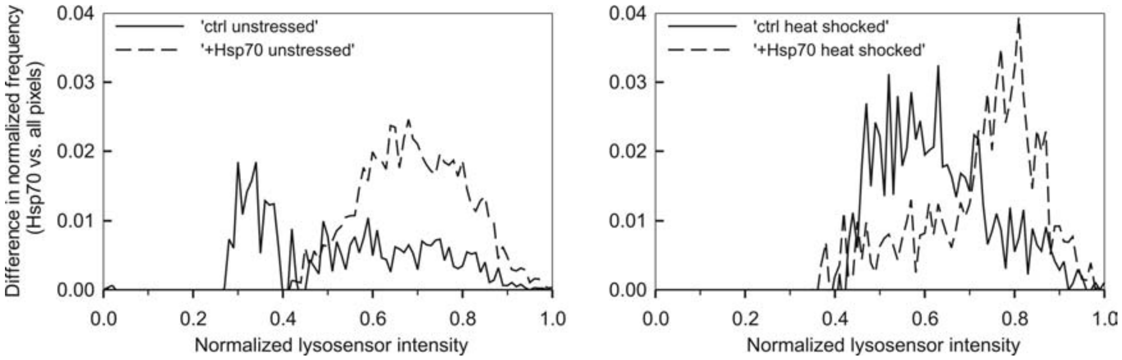

Fig. (3S). Preferential accumulation of surface labeled Hsp70 in acidic compartments Cells were stained and analyzed as shown in Fig. 5B. Difference histograms of Hsp70 positive and all pixels for each cell type and condition are shown. Values indicate at which lysosensor intensities surface labeled Hsp70 accumulates. Note that lysosensor is a pH sensitive dye, therefore high lysosensor intensities (≥ 0.6) point to more acidic compartments, i.e. lysosomes.

1873-4286/13 $58.00+.00 © 2013 Bentham Science Publishers

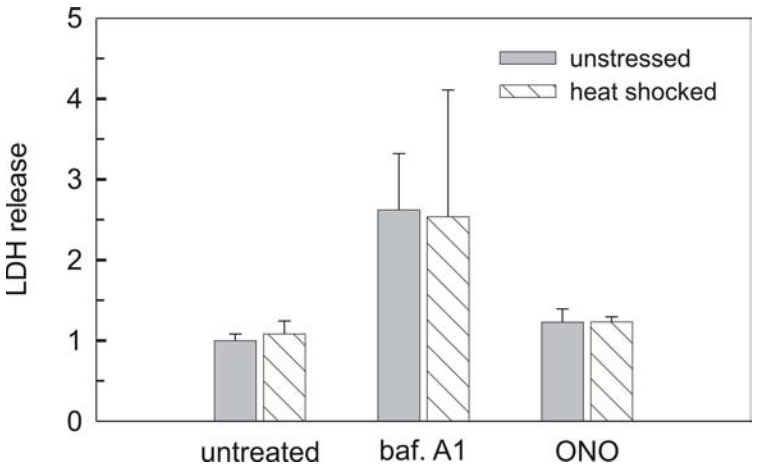

Fig. (4S). LDH activity of the supernatants of "+Hsp70" cells Adherent cells were incubated in the absence or presence of inhibitors at 37 ºC for 30 min, then either kept at 37 ºC or exposed to 43 ºC for 30 min. Cells were allowed to recover at 37 ºC for 30 min before measuring LDH activities. All samples were solubilized with detergent treatment before measurements. Note that released LDH activity from "untreated, unstressed" "+Hsp70" cells accounted for 0.18 % of total LDH activity of the cell lysate.

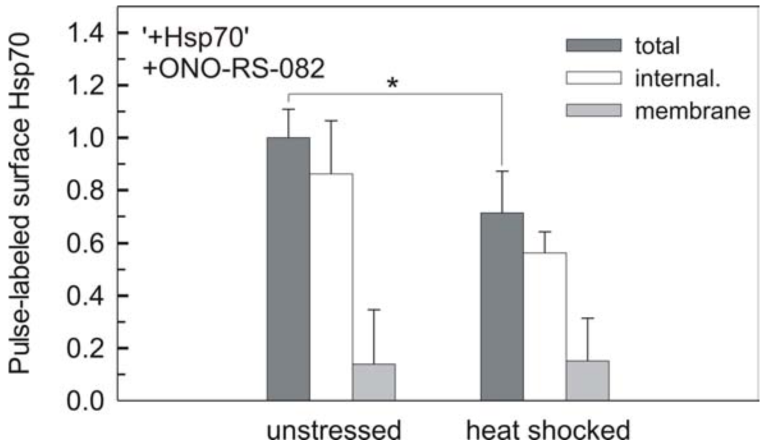

Fig. (5S). Effect of inhibiting endosomal recycling on the loss of pulse-labeled surface Hsp70 from heat stressed "+Hsp70" cells Cells were pulse-labeled with antibody at 37 ºC for 30 min, then incubated at 37 ºC or at 43 ºC for 30 min in the presence of ONO-RS-082. Total, internalized and membrane signals were assessed in fluorescence quenching experiments.

Movie 1 Mobility of internalized surface Hsp70 in the endolysosomal system Adherent "ctrl" cells were stained with αcmHsp70.1-AlexaFluor647 at 37 °C for 30 min and labeled with LysoTracker Green. Dynamic localization of the internalized surface Hsp70 (red) and the endolysosomal marker (green) was followed by dual color real time fluorescence microscopy. Actual time of recording is indicated in each frame, the movie is playing at 10 fps (image width= 61.5 μm).
